# Effects of substituting TV-watching time with physical activities or sleep on incident major depression. Results from the lifelines cohort study

**DOI:** 10.1192/j.eurpsy.2025.10045

**Published:** 2025-05-30

**Authors:** Rosa Palazuelos-González, Richard C. Oude Voshaar, Aart C. Liefbroer, Nynke Smidt

**Affiliations:** 1 Department of Epidemiology, University of Groningen, University Medical Center Groningen, Groningen, The Netherlands; 2 Department of Psychiatry, University of Groningen, University Medical Center Groningen, Groningen, The Netherlands; 3 Netherlands Interdisciplinary Demographic Institute (NIDI), Royal Netherlands Academy of Sciences (KNAW), The Hague, The Netherlands; 4 Department of Sociology, Vrije Universiteit Amsterdam (VU), Amsterdam, The Netherlands

**Keywords:** compositional data analysis, depression, physical activity, sedentary behavior, sleep

## Abstract

**Background:**

Physical activity is a known protective factor against depression but physical activity competes with other time-consuming behaviors that may increase depression risk. This study investigates the association between time spent in various movement-related activities and incident major depression, with a particular focus on the effects of replacing TV-watching time with other activities. Additionally, we explored whether the impact of substituting TV-watching differs across age groups.

**Methods:**

A population-based cohort study (Lifelines) with four-year follow-up, including 65,454 non-depressed adults (18+). Participants self-reported time spent in active commuting, leisure, sports, household, work or school physical-related activities, TV-watching, and sleep. Major depressive disorder was assessed using the Mini International Neuropsychiatric Interview. Compositional isotemporal data analysis was performed to analyze the effect of reallocating time in TV-watching with other activities adjusting for potential confounders. Interactions with age groups were also examined.

**Results:**

The incidence of major depressive disorder was 2.4%. Reallocating TV-watching time to any other physical activity or sleep reduced this risk in middle-aged adults. In older adults, only substituting TV-watching time with sports reduced the probability of becoming depressed. No significant reduction in probabilities for incident depression was found in younger adults.

**Conclusion:**

Replacing TV-watching time with other activities, including sleep, may serve as a preventive strategy against depressive disorder in middle-aged adults, while only the substitution with sports seems beneficial for older adults. Future research should aim to identify other activities, particularly in younger adults, that may prevent depression.

## Introduction

Depression is the leading contributor to global disability, affecting mental and physical health [1, 2]. Increasing physical activity is a promising prevention strategy [3]. It is estimated that 11.5% of depression cases could potentially be avoided if inactive adults meet the recommended 150 minutes of moderate to vigorous physical activity per week [4]. However, because time in a day is finite, spending more time in physical activity comes at the expense of other movement behaviors. It remains unclear whether reallocating time from potentially detrimental sedentary activities, such as TV-watching, to more beneficial behaviors like physical activity or sleep can reduce depression risk.

Most research on physical activity and depression has focused on intensity, without taking into account differences between types of activities. The physical activity paradox suggests that not all types of physical activity have equally beneficial effects on health [5, 6]. For example, studies found that leisure and/or transportation physical activity was associated with fewer depressive symptoms [5, 7], while others reported favorable associations for any type of physical activity [8]. This paradox may also extend to sedentary behavior. Mentally passive activities like TV-watching have been associated with an increased depression risk [9-11], possibly due to dopamine dysregulation, increased inflammation, greater consumption of unhealthy foods (partly driven by exposure to advertisements and “mindless eating”), as well as psychosocial factors like loneliness and social isolation [12-15]. A recent meta-analysis found that each additional hour of TV-watching was associated with a 5% higher risk of depression [11]. Substituting TV-watching time with brisk walking has also been linked to a reduced risk of depression [16]. Therefore, targeting TV-watching, rather than total sedentary time, may offer a more specific and effective basis for interventions.

Time spent in different types of activities changes across the life course. Young adults typically engage less in sedentary behavior and more in physical activities than middle-aged and older adults [17, 18]. Sleep duration also varies with age [19]. Moreover, the underlying mechanisms of depression may differ by age group. In late-life depression (≥60 years), factors like neurodegeneration and cerebrovascular disease, both influenced by physical activity, may become more prominent [20, 21]. Exploring how movement behaviors interact with age could inform age-specific prevention strategies.

Despite growing recognition of movement behaviors in mental health research, they are often studied without considering their temporal interrelation. Since days are fixed at 24 hours, time spent in one activity reduces the time available for others. This can be examined with compositional data analysis (CODA), a method increasingly used in time-use epidemiology to account for the proportional nature of mutually exclusive behaviors [22]. Most studies using CoDA have cross-sectional designs [23-28], although a few applied a prospective design [29, 30]. These studies generally suggest that replacing sedentary behavior with moderate-to-vigorous physical activity reduces depression risk, but evidence remains inconclusive.

This study addresses gaps in the current literature by applying a longitudinal design within the Lifelines Cohort Study to investigate how reallocating time from TV-watching to various types of physical activities and sleep affects major depression (MD) incidence. We also explore whether this association is moderated by age.

## Methods

**Figure 1.**
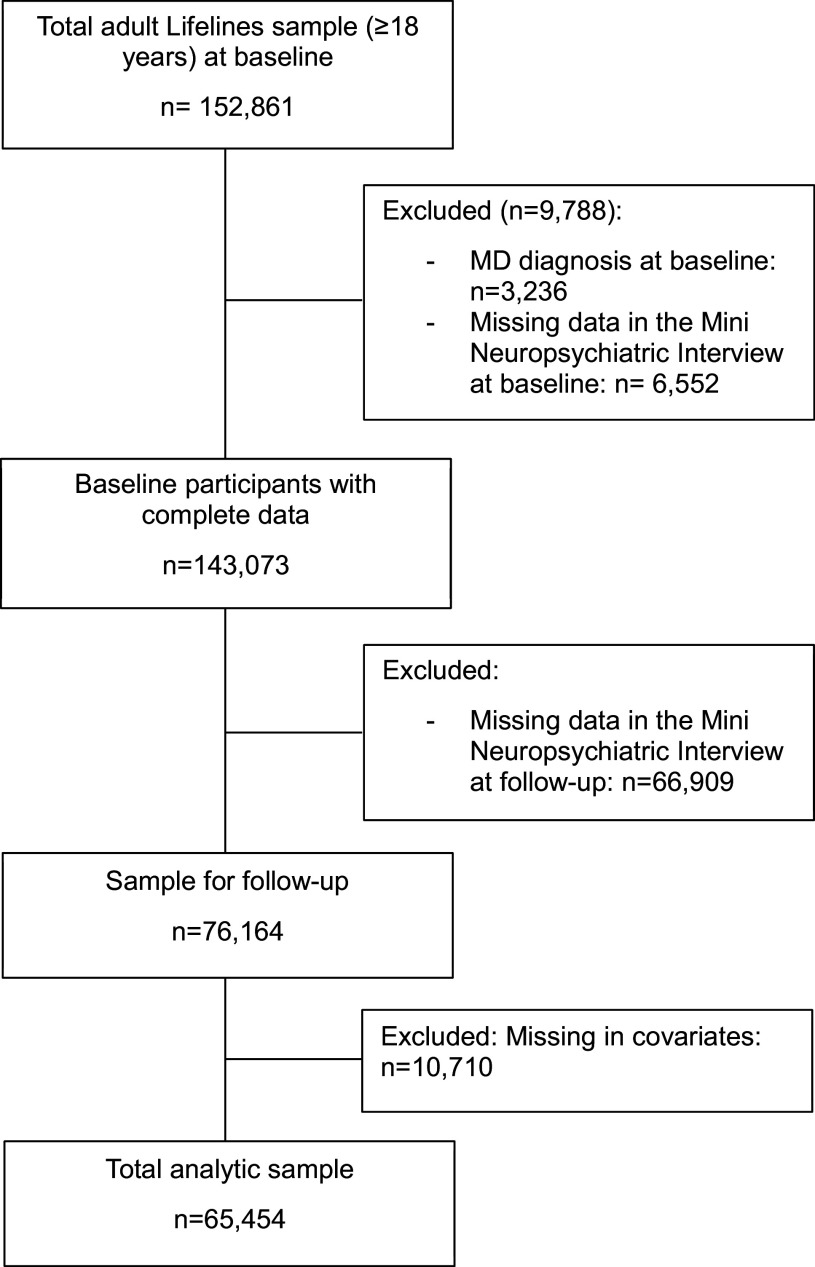
Flow chart of selected participants.

The Lifelines Cohort Study is a multi-disciplinary prospective population-based cohort study examining in a unique three-generation design the health and health-related behaviors of 167,729 persons living in the North of the Netherlands. It employs a broad range of investigative procedures in assessing the biomedical, socio-demographic, behavioral, physical, and psychological factors that contribute to the health and disease of the general population, with a special focus on multi-morbidity and complex genetics [31]. For the current study, we used baseline data collected between 2006 and 2013, and second wave data collected between 2014 and 2017. The selected sample includes adult participants (≥18 years) without an MD diagnosis at baseline, who participated in the second wave and had completed the Mini International Neuropsychiatric Interview. We excluded participants with missing values in covariates. The final sample included 65,454 participants (Figure 1).

The Lifelines Cohort Study adheres to the Declaration of Helsinki. The protocol was approved by the Medical Ethical Committee of the University Medical Center Groningen (number 1007/152). All participants provided written informed consent.

### Movement activities

TV-watching was assessed with the question: “On average, how much time (hours and/or minutes) per day do you spend watching TV?” This question has demonstrated moderate test–retest reliability (ICC 0.5–0.8) [32].

The Short QUestionnaire to ASsess Health-enhancing physical activity (SQUASH) was used to assess physical activities [33]. The SQUASH is a Dutch questionnaire developed to assess habitual physical activity levels and compliance with physical activity guidelines. Participants reported days per week in the past months and time per day (hours and/or minutes) spent on average in different types of activities, including household, sports, leisure, commuting, and activity at work and school. Total time (minutes/day) was obtained after processing the data as recommended [34]. Sports were classified as structured leisure-time activities with a Metabolic Equivalent Task ≥2 [35]. Given the small amount of time participants reported spending on active commuting, commuting was included in the leisure category. Final types of physical activity that were included in the analysis were household, sports, leisure/commuting, and activity at work and school. The SQUASH has demonstrated acceptable validity and reliability, with a reproducibility correlation of 0.58 and test–retest reliability between 0.6 and 0.8 [36, 37].

Lastly, sleep duration was determined using the question: “On average, how many hours and/or minutes do you sleep per full day (24 hours)?,” which has demonstrated good reliability (ICC 0.6–0.7) [38] and moderate validity against actigraphy (r ≈ 0.3–0.5) [39, 40].

### Major depression

Current major depressive disorder according to DSM-IV criteria was assessed with the Mini International Neuropsychiatric Interview (MINI) [41, 42]. It assesses two core symptoms, consistently depressed or down and anhedonia. It also evaluates seven related symptoms: loss of appetite, sleep disturbances, changes in movement speed, fatigue, feelings of worthlessness and guilt, difficulty concentrating or making decisions, and suicidal thoughts. Major depression (MD) is diagnosed when at least one core symptom and a total of five symptoms have been consistently present over the past two weeks. The MINI has a sensitivity of 0.94–0.95 and a specificity of 0.79–0.88 [43, 44].

### Covariates

For covariate selection, we developed a Directed Acyclic Graph based on prior evidence and theoretical considerations (Supplementary Material 1). Given that many health and lifestyle factors potentially lie on the causal pathway between movement behaviors and depression, we chose to adjust only for key sociodemographic variables to avoid overadjustment. These included age, sex, level of education (low, middle, and high based on obtained years of education), equivalized household income categorized in quartiles (including a dummy for those who did not provide information), and employment status (employed, non-employed, and retired). We also adjusted for the time (months) between baseline and follow-up interviews.

### Statistical analysis

Descriptive statistics are presented as frequencies for categorical variables and as mean with standard deviations for continuous variables. Differences between age groups were tested with chi-square and ANOVA tests.

To examine time spent in movement activities jointly, we applied compositional data analysis (CoDA). This method treats each movement activity as part of a constrained 24-hour composition by transforming each activity into an isometric log ratio [22]. Analyses were conducted in R 4.3.3 using the ‘compositions’ package. As 60% of participants reported zero minutes/day in certain activities, we imputed these values by using the log-ratio expectation–maximization algorithm as recommended [45] via the ‘zCompositions’ package [46]. After imputation, we calculated the distribution of time spent on each activity. In CoDA, this distribution is based on the geometric means of the time-use categories rather than the arithmetic mean [22]. Next, we performed multivariable binomial logistic regression analyses to examine the relationship between the movement activity composition and major depression incidence. We used a likelihood ratio test between nested logistic regression models to assess whether adding the movement activity composition to the model led to a statistically significant improvement of the model. Subsequently, we performed compositional isotemporal substitution analyses to reallocate 30, 60, 90, and 120 minutes/day from TV-watching time to either an equally divided proportion across all other behaviors or to a specific movement activity (1:many and 1:1 reallocations, respectively). The consequences of specific reallocations of TV watching time were expressed as changes in the predicted probability of incident MD, consistent with best practices for interpreting logistic models [47, 48]. We used 1,000 bootstrap repetitions to test the significance of differences in predicted probabilities between time-reallocated and original compositions.

We tested interactions between movement activity composition and age group (18–39, 40–59, ≥60 years) using a likelihood ratio test. When the inclusion of the interaction terms significantly improved the model fit (<0.05), we conducted stratified analyses by age group.

We conducted two sets of sensitivity analyses. The first addressed the self-reported nature of time-use data by performing CoDA exclusively among participants who reported a total time ≤ 1440 minutes/day, ensuring the plausibility of the 24-hour composition. The second repeated the CoDA using only participants who did not report zero time spent in any movement activity, to assess potential selection bias if the main analysis had been restricted to this subgroup.

Detailed information on the analytical procedures used can be found in Supplementary Material 2.

## Results

In the total sample (*n* = 65,454), the mean age was 44.8 years (±11.9) and 59.7% were women (Table 1). The mean follow-up time was 3.8 years, and 2.4% developed MD. Age group distributions were as follows: 31.4% younger, 56.1% middle, and 12.5% older adults.

**Table 1.** Descriptive characteristics of the analytic sample at baseline

	Total *n* = 65,454	Young adults *n* = 20,573	Middle-aged adults *n* = 36,722	Older adults *n* = 8,159	*p*-value
Age at baseline	44.8 ± 11.9	31.2 ± 6.0	47.9 ± 5.2	64.8 ± 4.2	<0.001
Sex					
Female	60	61	60	52	
Male	40	39	40	48	
Education					<0.001
Low	27	14	29	48	
Middle	41	46	42	24	
High	33	39	30	28	
Equivalized household income					<0.001
<1230	22	26	22	8	
1230- < 1590	19	20	20	18	
1590- < 1900	24	20	22	27	
> = 1900	21	19	18	41	
Not provided info	14	12	15	16	
Employment status					<0.001
Employed	67	69	75	22	
Retired	17	10	11	59	
Unemployed	17	21	14	18	
Incident major depression	2.4	2.9	2.4	1.0	<0.001

*Note:* Data is presented in mean ± standard deviation for continuous variables, or as frequencies for categorical variables.

The distribution of time spent in different movement activities is presented in Table 2, using both arithmetic and geometric means. As CoDA relies on the geometric mean composition, we focus our interpretation on that measure. Sleep was the activity with the highest proportion in the whole composition in the total sample, followed by leisure/commuting activities and TV-watching. The activity with the lowest proportion was sports.

**Table 2.** Compositional arithmetic and geometric mean (%) of time spent in movement activities

	TV-watching	Household	Sports	Work/school	Leisure/ commuting	Sleep
Arithmetic mean						
Total sample	12.11%	15.27%	5.10%	15.07%	15.41%	37.03%
Young adults	11.44%	15.64%	5.69%	16.94%	12.22%	38.07%
Middle-aged adults	11.88%	15.33%	4.77%	16.23%	15.79%	36.01%
Older adults	14.87%	14.10%	5.12%	5.11%	21.77%	39.03%
Geometric mean						
Total sample	15.27%	14.30%	0.39%	0.77%	15.84%	53.44%
Young adults	14.55%	15.58%	0.60%	1.32%	12.09%	55.87%
Middle-aged adults	15.05%	14.57%	0.33%	1.07%	16.87%	52.12%
Older adults	17.29%	9.97%	0.26%	0.04%	22.15%	50.28%

### Associations between movement activity composition and major depression incidence

The logistic regression model including the movement activity composition significantly improved model fit (Supplementary Table 7). The isometric log ratio for TV-watching time, compared to other activities, increased the odds of incident MD (Supplementary Table 9). In the total sample, reducing TV-watching time by 60 minutes and proportionally reallocating it to other activities decreased the likelihood of developing MD from 2.38 to 2.13%, representing a 10.72% reduction (Table 3). For 90- and 120-minute reallocations, significant reductions of 16.78 and 25.91% were observed, respectively.

**Table 3.** 1:Many time reallocations of TV-watching with the other activities

	Total sample			Young adults			Middle-aged adults			Older adults		
	Probability	Mean difference	% Likelihood reduction	Probability	Mean difference	% Likelihood reduction	Probability	Mean difference	% Likelihood reduction	Probability	Mean difference	% Likelihood reduction
Composition without reallocation	0.0238	-	-	0.0293	-	-	0.0239	-	-	0.0101	-	-
30 min/day	0.0229	−0.0010	−4.05	0.0295	0.0002	0.59	0.0220	−0.0018	−7.65	0.0095	−0.0006	−5.78
60 min/day	0.0213	−0.0026*	**−10.72**	0.0298	0.0005	1.71	0.0194	−0.0045*	**−18.78**	0.0087	−0.0014	−13.82
90 min/day	0.0198	−0.0040*	**−16.78**	0.0301	0.0008	2.72	0.0170	−0.0069*	**−28.85**	0.0079	−0.0022	−21.78
120 min/day	0.0176	−0.0062*	**−25.91**	0.0306	0.0013	4.30	0.0136	−0.0102*	**−42.92**	0.0067	−0.0033	−32.87

Values marked with * and in bold *p* < 0.05. Estimates are adjusted for covariates.

Table 4 presents the results of reallocating time from TV-watching to specific activities. Reallocating 30 minutes to sports or leisure/commute physical activities significantly reduced the probability of incident MD by 14.16 and 4.87%, respectively, with stronger effects observed at higher reallocation durations. Reallocating 60 minutes of TV-watching to sleep also reduced the probability of incident MD, from 2.38 to 2.11%. Additionally, reallocating 120 minutes of TV-watching to work/school-related activities was associated with a significant reduction of 24.70% in the likelihood of developing MD.

**Table 4.** 1:1 time reallocations of TV-watching with specific movement activities

	Total sample			Young adults			Middle-aged adults			Older adults		
	Probability	Mean difference	% Likelihood reduction	Probability	Mean difference	% Likelihood reduction	Probability	Mean difference	% Likelihood reduction	Probability	Mean difference	% Likelihood reduction
Composition without reallocation	0.0238	-	-	0.0293	-	-	0.0239	-	-	0.0101	-	-
*30 min less in TV-watching*												
Household	0.0234	−0.0004	−1.88	0.0297	0.0004	1.33	0.0227	−0.0012	−5.00	0.0097	−0.0003	−3.03
Sports	0.0205	−0.0034*	**−14.16**	0.0277	−0.0016	−5.37	0.0195	−0.0043*	**−18.08**	0.0071	−0.0030*	**−29.43**
Work/school	0.0230	−0.0008	−3.41	0.0303	0.0010	3.48	0.0214	−0.0024*	**−10.21**	0.0118	0.0018	17.49
Leisure/commute	0.0227	−0.0012*	**−4.87**	0.0294	0.0001	0.36	0.0219	−0.0019*	**−8.09**	0.0088	−0.0012	−11.99
Sleep	0.0228	−0.0011	−4.40	0.0295	0.0002	0.60	0.0218	−0.0020*	**−8.45**	0.0095	−0.0005	−5.27
*60 min less in TV-watching*												
Household	0.0220	−0.0018	−7.55	0.0301	0.0008	2.80	0.0203	−0.0036*	**−14.98**	0.0091	−0.0010	−9.92
Sports	0.0188	−0.0050*	**−21.04**	0.0278	−0.0015	−5.10	0.0170	−0.0068*	**−28.61**	0.0063	−0.0037*	**−37.17**
Work/school	0.0215	−0.0023	−9.80	0.0308	0.0014	4.91	0.0189	−0.0050*	**−20.82**	0.0112	0.0011	10.94
Leisure/commute	0.0211	−0.0028*	**−11.72**	0.0297	0.0004	1.34	0.0193	−0.0046*	**−19.10**	0.0079	−0.0022	−21.55
Sleep	0.0211	−0.0027*	**−11.34**	0.0298	0.0005	1.73	0.0191	−0.0048*	**−20.08**	0.0088	−0.0013	−12.90
*90 min less in TV-watching*												
Household	0.0207	−0.0031	−13.01	0.0305	0.0012	4.04	0.0180	−0.0058*	**−24.45**	0.0083	−0.0017	−17.18
Sports	0.0174	−0.0064*	**−26.86**	0.0280	−0.0013	−4.60	0.0149	−0.0090*	**−37.73**	0.0056	−0.0044*	**−43.81**
Work/school	0.0201	−0.0037	−15.66	0.0311	0.0018	6.10	0.0166	−0.0072*	**−30.38**	0.0103	0.0003	2.94
Leisure/commute	0.0196	−0.0043*	**−17.82**	0.0300	0.0007	2.26	0.0169	−0.0069*	**−29.01**	0.0070	−0.0030	−30.14
Sleep	0.0197	−0.0042*	**−17.58**	0.0301	0.0008	2.74	0.0166	−0.0073*	**−30.45**	0.0080	−0.0021	−20.55
*120 min less in TV-watching*												
Household	0.0186	−0.0052	−21.95	0.0310	0.0017	5.80	0.0146	−0.0092*	**−38.65**	0.0072	−0.0028	−28.13
Sports	0.0155	−0.0084*	**−35.14**	0.0283	−0.0010	−3.43	0.0119	−0.0120*	**−50.16**	0.0048	−0.0053	−52.24
Work/school	0.0180	−0.0059*	**−24.70**	0.0316	0.0023	7.83	0.0134	−0.0105*	**−43.96**	0.0091	−0.0010	−9.95
Leisure/commute	0.0174	−0.0064*	**−26.89**	0.0304	0.0011	3.76	0.0136	−0.0102*	**−42.93**	0.0059	−0.0041	−41.07
Sleep	0.0175	−0.0064*	**−26.78**	0.0306	0.0013	4.33	0.0132	−0.0106*	**−44.50**	0.0069	−0.0032	−31.49

*Note:* Values marked with * and in bold indicate *p* < 0.05. Estimates are adjusted for covariate

### Age-specific analyses

The logistic regression model including interactions between the activity composition and age group significantly improved model fit (Supplementary Table 11).

In young adults, reallocating TV-watching time to one or multiple movement activities did not significantly change predicted probabilities of incident MD (Tables 3 and 4).

Among middle-aged adults, reallocating 60 minutes from TV-watching to other activities significantly decreased the probability of incident MD from 2.39 to 1.94% (18.78% reduction). Reallocating 90 minutes resulted in a 28.85% reduction, and 120 minutes led to a 42.92% reduction. All reallocations of TV-watching time to specific activities were associated with reduced depression risk, except for reallocating only 30 minutes to household activities, which did not yield a significant effect. When reallocating 30 minutes specifically to sports, the reduction was 18.08%; to work/school physical activities, 10.21%; to leisure/commute activities, 8.09%; and to sleep, 8.45%. Reallocations to sports, at any given duration, resulted in the largest reductions in the probability of incident MD compared to all other activities.

In older adults, reallocating TV-watching time proportionally to other activities did not lead to statistically significant reductions in incident MD. Only reallocating TV-watching time to sports significantly reduced MD incidence, from 1.01 to 0.71% with 30 minutes, 0.63% with 60 minutes, and 0.56% with 90 minutes.

### Sensitivity analyses

Around 70% of the sample reported ≤1440 minutes/day. Compared to the full analytic sample, the geometric mean for sleep was notably higher in this subgroup. Associations were in the same direction as in the full sample but attenuated (Supplementary Tables 20 and 21). In 1:1 reallocations, the attenuation was particularly pronounced when TV-watching time was reallocated to sleep.

Among participants with non-zero values for all movement behaviors, the incidence of MD was 1.98%. Time spent on TV-watching and sleep was lower, while time spent on physical activities was higher compared to the full sample (Supplementary Table 23). In the full sample, reallocating 30 minutes of TV-watching time to only sports or leisure/commuting activities was associated with a decreased risk of incident MD, whereas among participants with non-zero values reallocating 30 minutes of TV-watching time to any other activity decreased MD. For example, reallocating TV-watching time to household activities reduced the incidence of MD from 2.38 to 2.34% (a 1.88% reduction) in the full sample, compared to a reduction from 1.98 to 1.74% (a 12.16% reduction) in the non-zero subgroup (Supplementary Tables 24 and 25).

## Discussion

The primary aim of this study was to examine the theoretical effects of reallocating time spent TV-watching to other movement behaviors or sleep within the total sample. Overall, replacing 60 minutes or more of TV-watching with other activities was associated with a reduced probability of developing MD. Notably, reallocating even 30 minutes of TV-watching to sports or leisure/commuting-related physical activity was linked to a lower predicted probability of incident MD. A similar reduction in MD risk was observed when 60 minutes of TV-watching was replaced with sleep.

Next, we examined whether the findings were uniform across all age groups. Clear age differences emerged. Younger adults did not appear to benefit from reallocating TV-watching time to other activities. Several explanations are possible. First, other risk factors may outweigh the impact of movement activities in this group. For example, genetic predisposition and childhood abuse may have a greater effect on early-onset depressive disorder than late-onset depression, thereby overruling other influences [49, 50]. Second, physical inactivity may need to persist longer to increase depression risk. Many sequelae of low physical activity, such as pro-inflammatory states or reduced BDNF expression, may increase depression risk progressively with duration and severity. [51–53]. Third, this group appears more active than older age groups, possibly having already surpassed the physical activity threshold protective against depression [54].

Middle-aged adults in this study showed the greatest benefits from reallocating TV-watching time. Even more realistic changes, like reallocating TV-watching by 30–60 minutes/day to one or multiple activities, were associated with reduced depression risk. Interestingly, replacing just 30 minutes of TV-watching with sports reduced depression risk nearly as much as redistributing 60 minutes across all activities. These findings align with those of Kandola et al., who reported that replacing 60 minutes of sedentary behavior with light or moderate physical activity, or with sleep, was associated with fewer depressive symptoms [29].

As in other research [55], the prevalence of depressive disorder was lowest in the oldest age group. Among older adults, only reallocating TV-watching time to sports reduced MD incidence. Two explanations may help explain this. First, the amount (or intensity) of physical activity required to prevent depression might increase with age [56], and sports, unlike other activity types in our study, are more likely to involve higher-intensity movement. Second, sports were the only physical activity we included typically performed in social groups. In later life, loneliness is a significant risk factor for depression [57], so the social aspect of sports may offer added protection. Furthermore, although reductions in MD incidence were also observed when reallocating time to leisure/commuting activities, these did not reach statistical significance, likely due to limited statistical power. Confirming these findings in a larger sample of older adults would be valuable, as it may help identify a broader range of effective activity types for reducing the risk of MD in later life. Substituting TV-watching time by sleep reduced MD incidence only in middle-aged adults, possibly reflecting unmet needs due to the high work-related demands and time pressure experienced by middle-aged adults. In older adults, the observed reductions in MD incidence were smaller than those seen in middle-aged adults and did not reach statistical significance. This may indicate a more limited effect of sleep reallocation in later life. A previous study restricted to older adults found that replacing sedentary time with sleep was even associated with worsened depression scores [30]. Although both insufficient and excessive sleep has been linked to increased depression risk, potentially through mechanisms such as neuroinflammation, hyperactivation of the hypothalamicpituitary–adrenal (HPA) axis, raising cortisol levels, and increased fatigue, and reduced motivation [58, 59], future studies should explore these relationships further in the context of time-interrelated activities.

In sensitivity analyses limited to participants reporting ≤1440 minutes/day, results remained directionally consistent with the full sample but were slightly attenuated. In contrast, analyses restricted to participants with non-zero values for all activities showed larger reductions in MD risk when reallocating TV-watching time. Compared to the full analytic sample, this subgroup was more likely to engage in work/school-related activities and sports and tended to be younger, more highly educated, and more often employed. As such, they resemble the middle-aged subgroup in our study to quite an extent, and their results mirrored those of the middle-aged participants in the full sample. Given these differences, it is important to perform CoDA both with and without excluding cases with zero values in time-use categories, to evaluate the impact of such in−/exclusions. While this issue is less pronounced when using objective time-use data (e.g. accelerometers), it is particularly relevant in studies relying on self-reported measures, like ours. Given our broad age range, excluding cases with zero values in any of the time-use categories would have seriously reduced the sample size and limited the generalizability of our findings to a narrower, more selective population.

Strengths of our study were the large sample size which enabled age-stratified analyses, the length of follow-up covering almost four years, the assessment of the DSM criteria for MD, and the application of CoDA to account for the interdependence of time-use behaviors. Nonetheless, some limitations should be mentioned. First, TV-watching becomes less common in young adults and may be replaced by spending time online on digital devices [60, 61]. Second, movement behaviors were based on self-report data, which can lead to overestimation, particularly for activities with higher intensities, due to social desirability [62] and variability in sleep patterns [40]. Furthermore, the SQUASH may not capture all daily activities, as the total time does not always add up to the total of 1440 minutes/day (24 hours). Consequently, a variety of remaining potential activities could be associated with incident depression. Additionally, detailed information on the specific types of sports reported by participants was not available. The dropout rate is associated with various health indicators linked to depression (data not shown), suggesting the likelihood of attrition bias and this could have led to an underestimation of our effects. Finally, although meta-analyses found a U-shaped association between sleep and incident depression [55], we did not stratify by sleep duration to preserve statistical power across age groups.

## Clinical implications and conclusion

A realistic substitution of 30 or 60 minutes of TV-watching with sports was associated with the greatest reduction in MD incidence, but only in middle-aged and older adults. Among middle-aged adults, benefits were also seen when TV-watching time was reallocated to household, work-related activities, leisure/commuting, or sleep, but these effects were smaller. These findings support promoting diverse physical activities in this age group. Reducing TV time may be a particularly effective public health strategy for middle-aged and older adults. While no significant effects were found in young adults, encouraging an active lifestyle remains important, as early physical activity predicts future behavior [63].

## Supporting information

10.1192/j.eurpsy.2025.10045.sm001 Palazuelos-González et al. supplementary material Palazuelos-González et al. supplementary material

## Data Availability

Data may be obtained from a third party and is not publicly available. Researchers may apply for access to the Lifelines data through the Lifelines organization. Detailed information about the application procedure and conditions of use is available on their website (http://www.lifelines-biobank.com). Data for the current project can be accessed under project number OV20_00505.

## Supplementary material

The supplementary material for this article can be found at http://doi.org/10.1192/j.eurpsy.2025.10045.
