# Supplementary material for: Effects of substituting TV-watching time with physical activities or sleep on incident major depression. Results from the lifelines cohort study
Source: Eur Psychiatry. 2025 May 30;68(1):e73. doi: 10.1192/j.eurpsy.2025.10045 (PMC12303778; doi:10.1192/j.eurpsy.2025.10045)
Supplement: Palazuelos-González et al. supplementary material [file S092493382510045Xsup001.docx]

Supplementary Material 1- Directed acyclic graph of covariate relationships and mechanistic pathways

Supplementary Figure 1. Directed acyclic graph (DAG) illustrating the assumed causal relationships among covariates included in the analysis.


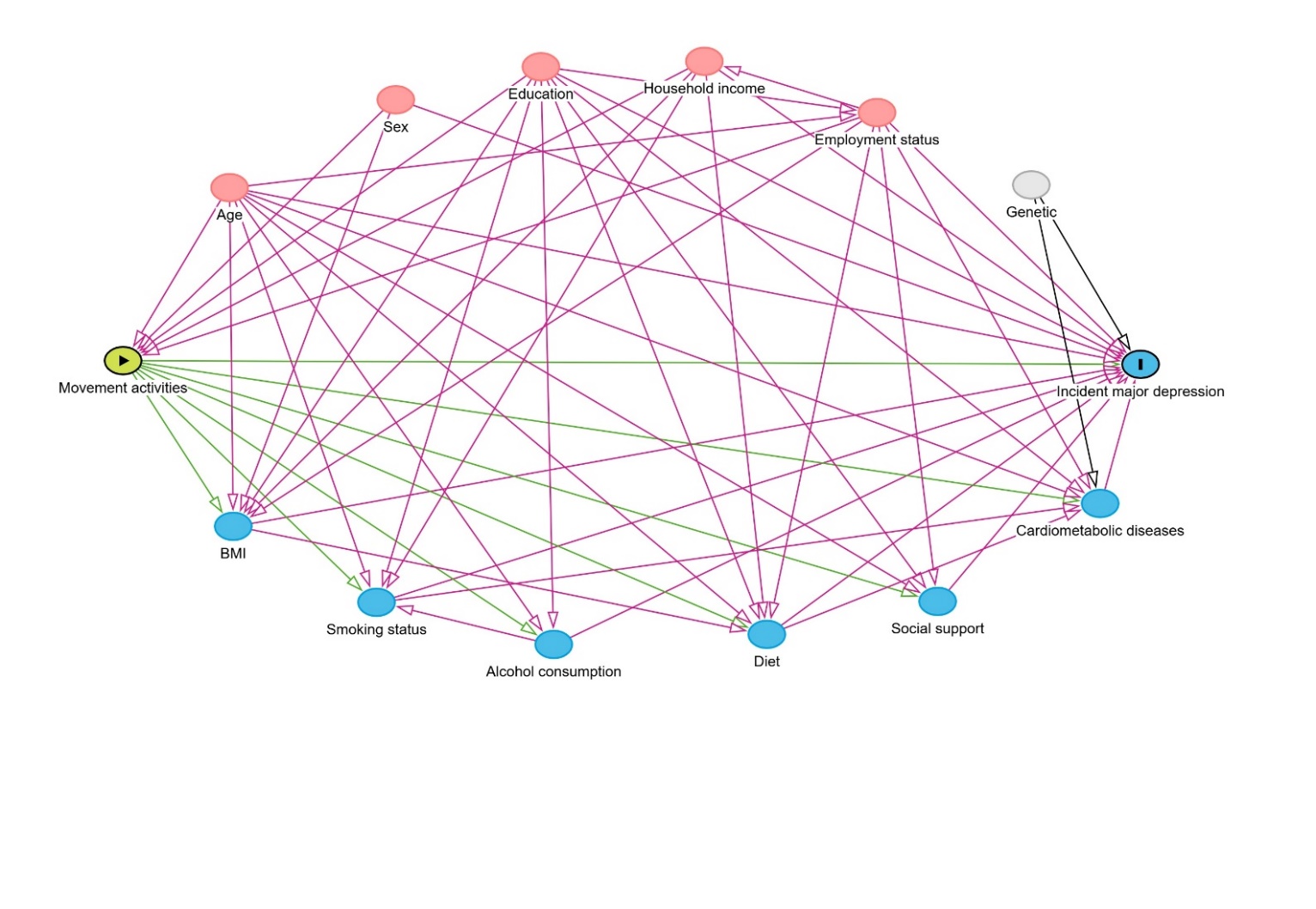
Node colors indicate:

Exposure variables


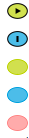

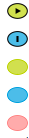

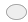


Outcome

Mediators

Potential confounders

Unobserved (latent) variable

Supplementary Table 1. Possible mechanisms between lifestyle and other variables to both physical activity and depression

|  | Mechanism |
| --- | --- |
| BMI | Inflammation, body perception, metabolic stress ^1, 2^ |
| Smoking status | Neurochemical, inflammatory, behavioral^3,4^ |
| Alcohol consumption | Neurochemical, inflammatory, behavioral^5,6^ |
| Diet | Nutritional status, inflammation, gut-brain axis^7,8^ |
| Cardiometabolic diseases | Biological stress, reduced quality of life, functional decline^9^ |
| Social support | Emotional support, resilience, social belonging^10,11^ |

References

1. Silveira EA, et al. Sedentary behavior, physical inactivity, abdominal obesity and obesity in adults and older adults: A systematic review and meta-analysis. Clin Nutr ESPEN. 2022;50:63-73. doi:10.1016/j.clnesp.2022.06.001
2. Jokela M, et al. Obesity as a causal risk factor for depression: Systematic review and meta-analysis of Mendelian Randomization studies and implications for population mental health. J Psychiatr Res. 2023;163:86-92. doi:10.1016/j.jpsychires.2023.05.034
3. Zhang J, et al. The association between different types of physical activity and smoking behavior. BMC Psychiatry. 2023;23(1):927. Published 2023 Dec 11. doi:10.1186/s12888-023-05416-1
4. Wootton RE, et al. Evidence for causal effects of lifetime smoking on risk for depression and schizophrenia: a Mendelian randomization study. Psychol Med. 2020;50(14):2435-2443. doi:10.1017/S0033291719002678
5. de Victo ER, et al. Association of physical activity and sitting time with tobacco and alcohol use in 222,495 adolescents from 66 countries. BMC Pediatr. 2024;24(1):596. Published 2024 Sep 19. doi:10.1186/s12887-024-05079-1
6. Li J, et al. Effect of alcohol use disorders and alcohol intake on the risk of subsequent depressive symptoms: a systematic review and meta-analysis of cohort studies. Addiction. 2020;115(7):1224-1243. doi:10.1111/add.14935
7. You Y, et al. Relationship between leisure-time physical activity and depressive symptoms under different levels of dietary inflammatory index. Front Nutr. 2022;9:983511. Published 2022 Sep 7. doi:10.3389/fnut.2022.983511
8. Lassale C, et al. Healthy dietary indices and risk of depressive outcomes: a systematic review and meta-analysis of observational studies [published correction appears in Mol Psychiatry. 2019 Jul;24(7):1094. doi: 10.1038/s41380-018-0299-7.] [published correction appears in Mol Psychiatry. 2021 Jul;26(7):3657. doi: 10.1038/s41380-021-01056-7.]. Mol Psychiatry. 2019;24(7):965-986. doi:10.1038/s41380-018-0237-8
9. Aune D, et al. Physical activity and the risk of type 2 diabetes: a systematic review and dose-response meta-analysis. Eur J Epidemiol. 2015;30(7):529-542. doi:10.1007/s10654-015-0056-z
10. White RL, et al. Physical activity and mental health: a systematic review and best-evidence synthesis of mediation and moderation studies. Int J Behav Nutr Phys Act. 2024;21(1):134. Published 2024 Nov 28. doi:10.1186/s12966-024-01676-6
11. Nouwen A, et al. Type 2 diabetes mellitus as a risk factor for the onset of depression: a systematic review and meta-analysis. Diabetologia. 2010;53(12):2480-2486. doi:10.1007/s00125-010-1874-x

Supplementary Material 2- Methodological appendix- Compositional Data Analysis

Contents

[**Introduction to compositional data analysis** 4](#_Toc197596529)

[**Steps for compositional data analysis** 4](#_Toc197596530)

[Step 1- Assessing data suitability for CoDA 4](#_Toc197596531)

[Step 2. Handling zeros 5](#_Toc197596532)

[Step 3. Descriptive statistics with compositional data analysis 6](#_Toc197596533)

[Step 4. Assessing the association between the movement activity composition and major depression 7](#_Toc197596534)

[Step 5. 1:many reallocations 7](#_Toc197596535)

[Step 6. 1:1 reallocations 9](#_Toc197596536)

[Step 7. Testing interactions for age groups 9](#_Toc197596537)

[*Young adults* 11](#_Toc197596538)

[*Midde-aged adults* 12](#_Toc197596539)

[*Older adults* 13](#_Toc197596540)

[Step 8. Sensitivity analysis 14](#_Toc197596541)

[≤24 hours of reported movement activities 14](#_Toc197596542)

[Participants without zero values 15](#_Toc197596543)

# **Introduction to compositional data analysis**

In time-use epidemiology, researchers examine how individuals spend time across a finite set of daily behaviors (typically physical activity, sedentary behavior, and sleep) and how these behaviors relate to health outcomes. Traditional statistical methods often treat these time-use components as independent variables, analyzing them in isolation or controlling for each other in regression models. However, this approach does not account for the co-dependency between time-use behaviors, as time spent in one behavior necessarily displaces time from others due to the fixed 24-hour day.

Compositional data analysis (CoDA) addresses this issue by recognizing that time-use data are compositional in nature. The parts, such as minutes spent in different activities, are proportions of a whole, consequently they are constrained and interrelated. CoDA methods account for this constraint and interdependence by using log-ratio transformations, which allow for interpretation of the relative distribution of time among behaviors (1-2). This approach ensures that inferences reflect the balance and trade-offs between activities, rather than treating them as absolute and unrelated quantities.

CoDA:

- Maintains the constant-sum constraint (e.g., 24 hours -1440min-)
- Accounts for the relative nature of time-use data
- Avoids issues like collinearity that arise in standard regression when modeling mutually exclusive behaviors
- Allows for isotemporal substitution modeling within a coherent statistical framework

In order to examine the aim of our study, we analyzed our data using CoDA. Below, we outline the methodological steps taken to implement CoDA in our study.

# **Steps for compositional data analysis**

## Step 1- Assessing data suitability for CoDA

Before conducting the CoDA, we first took a closer look at the variables we planned to include (TV-watching, household activities, sports, work and school, leisure and commuting, and sleep) to make sure they were appropriate for this type of analysis. CoDA works by calculating log-ratios between time spent in each activity, and this only works if there are no zeros in the data. We checked for any zero values in the dataset, since they would need to be handled before moving forward with the analysis.

Supplementary Table 2. Descriptive statistics and number of zero values for time spent in movement activities (minutes/day)

|  | Min | 1^st^ quartile | Median | Mean | 3^rd^ quartile | Max | Zero values |
| --- | --- | --- | --- | --- | --- | --- | --- |
| TV watching | 0 | 90 | 120 | 149.7 | 180 | 960 | 547 |
| Household | 0 | 90 | 180 | 202.9 | 270 | 1500 | 3522 |
| Sports | 0 | 0 | 60 | 66.36 | 115 | 1290 | 26978 |
| Work and school | 0 | 0 | 192 | 214.7 | 420 | 1344 | 25843 |
| Leisure/commuting | 0 | 90 | 170 | 207.4 | 285 | 1680 | 2125 |
| Sleep | 0 | 420 | 450 | 448 | 480 | 840 | 7 |

## Step 2. Handling zeros

Activities such as work and school or sports have the largest amount of zeros in our dataset. Nonetheless, all of them have zeros. Potential solutions to this problem are:

1. Excluding participants with zeros in the sample. This creates a highly selective and reduced sample (18,820 participant data compared to 65,454)
2. Replacing the zeros with a sensible value to produce a complete dataset, which can be done in three different ways(4):
   1. Simple: replaces zeros in each affected behavior with a fixed value and rescales the data to add up to the constant sum.
   2. Multiplicative: replaces zeros by fixed values, but the values in the observed (non-zero) behaviors are adjusted multiplicatively in a meaningful way to preserve the ratios between them, i.e. to not modify their relative relationships.
   3. Log-ration Expectation-Maximization (lrEM): this is a parametric method (i.e. it relies on a probabilistic model for the data) and, unlike the other approaches, makes use of the information about the co-dependence structure between behaviors to produce estimated values of the zeros.

Rasmussen *et al* compared the statistical properties of these three methods and found that the lrEM method outperformed the other replacement methods as it had the smallest influence on the structure of relative variation of the datasets (4). Therefore, using the *zCompositions* package in R (5), we imputed the zero values using the lrEM method. The descriptives of the movement activity variables after imputation are shown in Table 3.

Supplementary Table 3. Descriptive statistics for time spent in movement activities (minutes/day) after imputing zeros

|  | Min | 1^st^ quartile | Median | Mean | 3^rd^ quartile | Max | Zero values |
| --- | --- | --- | --- | --- | --- | --- | --- |
| TV watching | 0.778 | 90.0 | 120.0 | 149.7 | 180.0 | 960.0 | 0 |
| Household | 0.570 | 90.0 | 180.0 | 202.9 | 270.0 | 1,500.0 | 0 |
| Sports | 0.001 | 0.3 | 60.0 | 66.4 | 115.0 | 1,290.0 | 0 |
| Work and school | 0.001 | 0.2 | 192.0 | 214.8 | 420.0 | 1,344.0 | 0 |
| Leisure/commuting | 0.659 | 90.0 | 170.0 | 207.5 | 285.0 | 1,680.0 | 0 |
| Sleep | 1.000 | 420.0 | 450.0 | 448.0 | 480.0 | 840.0 | 0 |

In the imputed dataset, all zeros have been replaced. Median and mean values remain virtually unchanged.

## Step 3. Descriptive statistics with compositional data analysis

Using the *compositions* package from R, we group all movement activities and define that these activities are components of our composition. While presenting the arithmetic mean (e.g. TV watching time/ total time) provides a good idea of how participants use their time proportionally (Table 4), this approach does not account for the compositional nature of the data. Instead, the geometric mean (Table 5), calculated based on log-ratio-transformed data and then transformed into proportions, offers a more accurate summary of time-use patterns. The geometric mean differs for activities such as sports, work, and sleep, where variability across individuals is high. In these cases, the geometric mean reduces the influence of extreme values and better reflects the central tendency of the relative time use of the sample.

Supplementary Table 4. Arithmetic mean of time spent across activities

|  | TV watching | Household | Sports | Work/school | Leisure | Sleep |
| --- | --- | --- | --- | --- | --- | --- |
| Total sample  n= 65,454 | 12.11% | 15.27% | 5.10% | 15.07% | 15.41% | 37.03% |

Supplementary Table 5. Geometric mean composition of time spent across activities

|  | TV watching | Household | Sports | Work/school | Leisure | Sleep |
| --- | --- | --- | --- | --- | --- | --- |
| Total sample  n= 65,454 | 15.27% | 14.30% | 0.39% | 0.77% | 15.84% | 53.44% |

Moreover, in compositional analysis a meaningful estimation of the relative dispersion structure is estimated by what is called the variation matrix, which is a symmetric matrix that contains all the possible log-ratio variances. That is, the variances of the logarithms of all pair-wise ratios between parts. A value close to zero implies that the two parts involved in the ratio (arranged by rows and columns in the matrix) are highly proportional.

Supplementary Table 6. Variation matrix

|  | TV watching | Household | Sports | Work/school | Leisure/commuting | Sleep |
| --- | --- | --- | --- | --- | --- | --- |
| TV watching | 0.00 | 2.60 | 17.03 | 23.87 | 2.16 | 0.51 |
| Household | 2.60 | 0.00 | 18.25 | 25.27 | 3.65 | 2.17 |
| Sports | 17.03 | 18.25 | 0.00 | 37.79 | 18.13 | 16.40 |
| Work and school | 23.87 | 25.27 | 37.79 | 0.00 | 24.15 | 23.08 |
| Leisure/commuting | 2.16 | 3.65 | 18.13 | 24.15 | 0.00 | 1.67 |
| Sleep | 0.51 | 2.17 | 16.40 | 23.08 | 1.67 | 0.00 |

## Step 4. Assessing the association between the movement activity composition and major depression

CoDA allows to observe whether the model with and without the original composition has a significant effect on any outcome, in our case, incident major depression. To assess this, we used the likelihood ratio test.

Supplementary Table 7. Likelihood ratio test for logistic regression models without and with activity composition

|  | Log- likelihood | DF | Chi^2^ | Pr (>Chi^2^) |
| --- | --- | --- | --- | --- |
| Model 1- sex, age, income, education, employment and follow-up time | -7189.6 | 13 | - | - |
| Model 2- model 1 + transformed activity composition (5 ilr coordinates) | -7169.2 | 18 | 40.74 | <0.001 |

With a Chi-squared statistic of 40.74 and 18 degrees of freedom, and a p-value of <0.001, we conclude that the model including the activity composition significantly improves model fit.

## Step 5. 1:many reallocations

After confirming that the activity composition significantly contributes to predicting the incidence of major depression, we proceeded to examine the effects of reallocating time from TV watching to the other movement activities using 1:many substitutions. In this type of reallocation, we assessed the impact of reducing TV-watching by 30, 60, 90, and 120 minutes per day, with the removed time being proportionally distributed across all remaining behaviors. In doing so, an important question is how to deal with participants who spend less than the amount to be reallocated on TV watching. Rather than to remove these respondents from the analysis, we reallocated the maximum amount of time they could contribute (reducing their TV-watching time to just one minute to avoid introducing zeros into the composition) and redistributed the remainder proportionally across the other movement activities. For instance, if a participant watched 110 minutes/day of TV and we wanted to reallocate 120 minutes, we used the available 109 minutes, preserving 1 minute of TV-watching time.

We present the results of reallocating TV watching by predicted probabilities of incident major depression. This approach offers an intuitive interpretation of effects and is particularly suitable given the binary nature of our outcome. Moreover, predicted probabilities are increasingly recognized as the preferred output in logistic models, as they avoid the identification and scaling issues that come with raw coefficients of logit and probit models, giving a clearer picture of absolute risk changes (6). To estimate the consequences of these reallocations, we averaged the predicted probabilities of major depression incidence across all participants. In this way, we capture the heterogeneity of the sample and yield estimates that are more representative. In contrast, estimating predictions by centering covariates can produce results that do not reflect any actual individual and obscure the variation across the sample.

Our general approach to calculating these predicted probabilities consisted of five steps:

1. Calculate the predicted probability of incident depression in the original sample (base model)
2. Reallocate the time spent on activities as outlined above
3. Use the parameter estimates from the base model and calculate the predicted probability of incident depression in the dataset adjusted in step 2
4. Calculate the difference in predicted probabilities between steps 3 and 1
5. Use bootstrapping (1000 repetitions) to test whether the difference in predicted probabilities calculated in step 4 is statistically significant.

The results of the 1:many reallocation procedure are presented in Supplementary Table 8.

Supplementary Table 8. 1:many reallocations of TV watching time with the rest of the activities in the total sample

|  | Predicted prob | 95% CI | Mean difference | 95% CI | P-value | % likelihood in reduction |
| --- | --- | --- | --- | --- | --- | --- |
| Composition without reallocation | 0.0238 | (0.0191, 0.0286) |  |  |  |  |
| 30 min/day | 0.0229 | (0.0182, 0.0275) | -0.0010 | (-0.0017, -0.0003) | 0.0603 | -4.053 |
| 60 min/day | 0.0213 | (0.0166, 0.0260) | **-0.0026** | **(-0.0042, -0.0007)** | **0.0432** | -10.724 |
| 90 min/day | 0.0198 | (0.0150, 0.0247) | **-0.0040** | **(-0.0064, -0.0011)** | **0.0406** | -16.784 |
| 120 min/day | 0.0177 | (0.0126, 0.0228) | **-0.0062** | **(-0.0096, -0.0019)** | **0.0344** | -25.911 |

The estimated logistic regression model on which this 1:many (and the subsequent 1:1 reallocations) are based is presented in Supplementary Table 9.

SupplementaryTable 9. Estimates for the 1:many reallocations with TV-watching

|  | Estimate | Std. Error | z- value | Pr (>\|z\|) |
| --- | --- | --- | --- | --- |
| (Intercept) | -2.257232 | 0.193369 | -11.673 | <0.001 |
| iIr. TV-watching 1 | 0.134802 | 0.044024 | 3.062 | <0.001 |
| iIr. TV-watching 2 | 0.087069 | 0.024128 | 3.609 | 0. 000308 |
| iIr. TV-watching 3 | 0.010892 | 0.014643 | 0.744 | 0.45699 |
| iIr. TV-watching 4 | 0.059162 | 0.019654 | 3.01 | 0.002611 |
| iIr. TV-watching 5 | 0.056265 | 0.040708 | 1.382 | 0.166923 |
| Sex (men) | -0.161491 | 0.060427 | -2.672 | 0.007529 |
| Age at baseline | -0.022698 | 0.002451 | -9.262 | <0.001 |
| Income- 1230-<1590 | -0.218702 | 0.076021 | -2.877 | 0.004017 |
| Income- 1590-<1900 | -0.346615 | 0.080285 | -4.317 | <0.001 |
| Income- >=1900 | -0.280615 | 0.084727 | -3.312 | <0.001 |
| Income- Not provided info | -0.1813 | 0.080159 | -2.262 | 0.023713 |
| Middle education | -0.336378 | 0.061334 | -5.484 | <0.001 |
| High education | -0.643696 | 0.076572 | -8.406 | <0.001 |
| Retired | -0.009088 | 0.088436 | -0.103 | 0.918148 |
| Unemployed | 0.303004 | 0.065725 | 4.61 | <0.001 |
| Follow-up time | 0.000741 | 0.002421 | 0.306 | 0.759552 |

## Step 6. 1:1 reallocations

In this type of reallocation, our aim is to examine the impact of replacing TV-watching time using the same time intervals as in the 1:many reallocations. However, instead of distributing the reduced time proportionally across all remaining activities, the full amount is reassigned to a specific movement behavior (e.g., household or sports).

Supplementary Table 10. 1:1 reallocations of TV-watching in different amounts of time in the total sample

|  | Predicted prob | 95% CI | Mean difference | | 95% CI | P-value | % likelihood in reduction |
| --- | --- | --- | --- | --- | --- | --- | --- |
| Composition without reallocation | 0.0238 | (0.0191, 0.0286) |  | |  |  |  |
| 30 min less in TV watching | |  |  |  | |  |  |
| Household | 0.0234 | (0.0187, 0.0281) | -0.0004 | | (-0.0012, 0.0003) | 0.3283 | -1.878 |
| Sports | 0.0205 | (0.0163, 0.0246) | **-0.0034** | | **(-0.0044, -0.0023)** | **0.0000** | -14.164 |
| Work/school | 0.0230 | (0.0184, 0.0277) | -0.0008 | | (-0.0019, 0.0002) | 0.2421 | -3.415 |
| Leisure/commute | 0.0227 | (0.0181, 0.0272) | **-0.0012** | | **(-0.0019, -0.0004)** | **0.0337** | -4.869 |
| Sleep | 0.0228 | (0.0182, 0.0274) | -0.0011 | | (-0.0018, -0.0002) | 0.0650 | -4.405 |
| 60 min less in TV watching | |  |  |  | |  |  |
| Household | 0.0220 | (0.0172, 0.0269) | -0.0018 | | (-0.0034, 0.0000) | 0.1440 | -7.555 |
| Sports | 0.0188 | (0.0146, 0.0230) | **-0.0050** | | **(-0.0066, -0.0033)** | **0.0002** | -21.038 |
| Work/school | 0.0215 | (0.0168, 0.0263) | -0.0023 | | (-0.0041, -0.0003) | 0.0810 | -9.802 |
| Leisure/commute | 0.0211 | (0.0165, 0.0256) | **-0.0028** | | **(-0.0044, -0.0010)** | **0.0249** | -11.721 |
| Sleep | 0.0211 | (0.0164, 0.0259) | **-0.0027** | | **(-0.0044, -0.0007)** | **0.0464** | -11.340 |
| 90 min less in TV watching | |  |  |  | |  |  |
| Household | 0.0207 | (0.0158, 0.0257) | -0.0031 | | (-0.0056, -0.0002) | 0.1078 | -13.006 |
| Sports | 0.0174 | (0.0132, 0.0217) | **-0.0064** | | **(-0.0086, -0.0038)** | **0.0005** | -26.855 |
| Work/school | 0.0201 | (0.0153, 0.0250) | -0.0037 | | (-0.0062, -0.0008) | 0.0573 | -15.660 |
| Leisure/commute | 0.0196 | (0.0149, 0.0243) | **-0.0043** | | **(-0.0066, -0.0015)** | **0.0252** | -17.825 |
| Sleep | 0.0197 | (0.0148, 0.0245) | **-0.0042** | | **(-0.0068, -0.0012)** | **0.0429** | -17.581 |
| 120 min less in TV watching | |  |  |  | |  |  |
| Household | 0.0186 | (0.0133, 0.0239) | -0.0052 | | (-0.0087, -0.0008) | 0.0735 | -21.950 |
| Sports | 0.0155 | (0.0110, 0.0199) | **-0.0084** | | **(-0.0114, -0.0047)** | **0.0015** | -35.143 |
| Work/school | 0.0180 | (0.0128, 0.0231) | -0.0059 | | (-0.0092, -0.0014) | 0.0418 | -24.704 |
| Leisure/commute | 0.0174 | (0.0125, 0.0224) | **-0.0064** | | **(-0.0097, 0.0023)** | **0.0232** | -26.889 |
| Sleep | 0.0175 | (0.0124, 0.0226) | **-0.0064** | | **(-0.0099, -0.0019)** | **0.0355** | -26.784 |

## Step 7. Testing interactions for age groups

In our introduction, we provided reasons for why we potentially expect age differences in the relationship between movement activities and incidence of major depression. We performed a likelihood ratio test to test models without and with interaction between age and the activity composition to decide whether we should continue with a stratified analysis (18–39y for young adults, 40–59y for middle-aged adults, ≥60y for older adults). The results of these tests are presented in Supplementary Table 11.

Supplementary Table 11. Likelihood ratio test for logistic regression models without and with interaction terms with age groups

|  | Log- likelihood | DF | Chi^2^ | Pr (>Chi^2^) |
| --- | --- | --- | --- | --- |
| Model without interaction terms | -7169.2 | 18 |  |  |
| Model with interaction terms between age and activity composition | -7159.4 | 28 | 19.754 | 0.03166 |

After confirming that the model with interaction terms significantly improved model fit, we proceeded to conduct a stratified analysis by age group. The following tables show the results of this stratified analysis.

Supplementary Table 12. Mean composition (proportions) by age group (based on geometric means)

|  | TV watching | Household | Sports | Work/school | Leisure | Sleep |
| --- | --- | --- | --- | --- | --- | --- |
| Total sample | 15.27% | 14.30% | 0.39% | 0.77% | 15.84% | 53.44% |
| Young adults | 14.55% | 15.58% | 0.60% | 1.32% | 12.09% | 55.87% |
| Middle-aged adults | 15.05% | 14.57% | 0.33% | 1.07% | 16.87% | 52.12% |
| Older adults | 17.29% | 9.97% | 0.26% | 0.04% | 22.15% | 50.28% |

### Young adults

Supplementary Table 13. 1:many reallocations of TV watching time with the rest of the activities in young adults

|  | Predicted prob | 95% CI | Mean difference | 95% CI | P-value | % likelihood in reduction |
| --- | --- | --- | --- | --- | --- | --- |
| Composition without reallocation | 0.0293 | (0.0200, 0.0386) | - | - | - | - |
| 30 min/day | 0.0295 | (0.0198, 0.0391) | 0.0002 | (-0.0012, 0.0017) | 0.4951 | 0.591 |
| 60 min/day | 0.0298 | (0.0190, 0.0407) | 0.0005 | (-0.0030, 0.0050) | 0.4981 | 1.708 |
| 90 min/day | 0.0301 | (0.0182, 0.0420) | 0.0008 | (-0.0048, 0.0080) | 0.4988 | 2.720 |
| 120 min/day | 0.0306 | (0.0165, 0.0447) | 0.0013 | (-0.0072, 0.0131) | 0.5020 | 4.303 |

Supplementary Table 14. 1:1 reallocations of TV-watching in different amounts of time in young adults

|  | Predicted prob | 95% CI | Mean difference | 95% CI | P-value | % likelihood in reduction |
| --- | --- | --- | --- | --- | --- | --- |
| Composition without reallocation | 0.0293 | (0.0200, 0.0386) | - | - | - | - |
| *30 min less in TV watching* | |  |  |  |  |  |
| Household | 0.0297 | (0.0201, 0.0393) | 0.0004 | (-0.0011, 0.0020) | 0.4480 | 1.335 |
| Sports | 0.0277 | (0.0187, 0.0367) | -0.0016 | (-0.0037, 0.0006) | 0.2621 | -5.371 |
| Work/school | 0.0303 | (0.0205, 0.0402) | 0.0010 | (-0.0012, 0.0032) | 0.3739 | 3.479 |
| Leisure/commute | 0.0294 | (0.0200, 0.0389) | 0.0001 | (-0.0015, 0.0016) | 0.4969 | 0.359 |
| Sleep | 0.0295 | (0.0198, 0.0391) | 0.0002 | (-0.0013, 0.0018) | 0.4993 | 0.601 |
| *60 min less in TV watching* | |  |  |  |  |  |
| Household | 0.0301 | (0.0193, 0.0409) | 0.0008 | (-0.0029, 0.0052) | 0.4741 | 2.796 |
| Sports | 0.0278 | (0.0177, 0.0379) | -0.0015 | (-0.0051, 0.0028) | 0.4135 | -5.098 |
| Work/school | 0.0308 | (0.0196, 0.0419) | 0.0014 | (-0.0026, 0.0063) | 0.4487 | 4.913 |
| Leisure/commute | 0.0297 | (0.0191, 0.0403) | 0.0004 | (-0.0033, 0.0048) | 0.4953 | 1.342 |
| Sleep | 0.0298 | (0.0189, 0.0407) | 0.0005 | (-0.0033, 0.0055) | 0.5012 | 1.726 |
| *90 min less in TV watching* | |  |  |  |  |  |
| Household | 0.0305 | (0.0186, 0.0424) | 0.0012 | (-0.0045, 0.0083) | 0.4814 | 4.037 |
| Sports | 0.0280 | (0.0169, 0.0390) | -0.0013 | (-0.0067, 0.0055) | 0.4574 | -4.599 |
| Work/school | 0.0311 | (0.0189, 0.0433) | 0.0018 | (-0.0041, 0.0093) | 0.4713 | 6.104 |
| Leisure/commute | 0.0300 | (0.0183, 0.0417) | 0.0007 | (-0.0049, 0.0077) | 0.4950 | 2.257 |
| Sleep | 0.0301 | (0.0181, 0.0422) | 0.0008 | (-0.0050, 0.0087) | 0.5016 | 2.744 |
| *120 min less in TV watching* | |  |  |  |  |  |
| Household | 0.0310 | (0.0169, 0.0451) | 0.0017 | (-0.0069, 0.0134) | 0.4902 | 5.796 |
| Sports | 0.0283 | (0.0153, 0.0413) | -0.0010 | (-0.0088, 0.0101) | 0.4854 | -3.429 |
| Work/school | 0.0316 | (0.0172, 0.0460) | 0.0023 | (-0.0065, 0.0144) | 0.4885 | 7.830 |
| Leisure/commute | 0.0304 | (0.0166, 0.0442) | 0.0011 | (-0.0073, 0.0127) | 0.4981 | 3.761 |
| Sleep | 0.0306 | (0.0163, 0.0449) | 0.0013 | (-0.0076, 0.0141) | 0.5046 | 4.331 |

### Midde-aged adults

Supplementary Table 15. 1:many reallocations of TV watching time with the rest of the activities in middle-aged adults

|  | Predicted prob | 95% CI | Mean difference | 95% CI | P-value | % likelihood in reduction |
| --- | --- | --- | --- | --- | --- | --- |
| Composition without reallocation | 0.0239 | (0.0175, 0.0302) | - | - | - | - |
| 30 min/day | 0.0220 | (0.0161, 0.0280) | -0.0018 | (-0.0027, -0.0009) | 0.0068 | -7.653 |
| 60 min/day | 0.0194 | (0.0138, 0.0250) | **-0.0045** | **(-0.0061, -0.0024)** | **0.0022** | -18.781 |
| 90 min/day | 0.0170 | (0.0117, 0.0223) | **-0.0069** | **(-0.0091, -0.0037)** | **0.0018** | -28.855 |
| 120 min/day | 0.0136 | (0.0087, 0.0186) | **-0.0102** | **(-0.0131, -0.0058)** | **0.0009** | -42.925 |

Supplementary Table 16. 1:1 reallocations of TV-watching in different amounts of time in middle-aged adults

|  | Predicted prob | 95% CI | Mean difference | 95% CI | P-value | % likelihood in reduction |
| --- | --- | --- | --- | --- | --- | --- |
| Composition without reallocation | 0.0239 | (0.0175, 0.0302) | - | - | - | - |
| *30 min less in TV watching* | |  |  |  |  |  |
| Household | 0.0227 | (0.0166, 0.0287) | -0.0012 | (-0.0021, -0.0002) | 0.0833 | -4.999 |
| Sports | 0.0195 | (0.0142, 0.0249) | **-0.0043** | **(-0.0057, -0.0029)** | **0.0001** | -18.079 |
| Work/school | 0.0214 | (0.0157, 0.0272) | **-0.0024** | **(-0.0037, -0.0010)** | **0.0131** | -10.214 |
| Leisure/commute | 0.0219 | (0.0161, 0.0278) | **-0.0019** | **(-0.0028, -0.0010)** | **0.0043** | -8.089 |
| Sleep | 0.0218 | (0.0159, 0.0278) | **-0.0020** | **(-0.0030, -0.0009)** | **0.0080** | -8.447 |
| *60 min less in TV watching* | |  |  |  |  |  |
| Household | 0.0203 | (0.0145, 0.0260) | **-0.0036** | **(-0.0052, -0.0015)** | **0.0151** | -14.978 |
| Sports | 0.0170 | (0.0121, 0.0220) | **-0.0068** | **(-0.0086, -0.0048)** | **0.0000** | -28.610 |
| Work/school | 0.0189 | (0.0135, 0.0243) | **-0.0050** | **(-0.0066, -0.0028)** | **0.0013** | -20.819 |
| Leisure/commute | 0.0193 | (0.0138, 0.0248) | **-0.0046** | **(-0.0061, -0.0026)** | **0.0011** | -19.100 |
| Sleep | 0.0191 | (0.0135, 0.0246) | **-0.0048** | **(-0.0065, -0.0025)** | **0.0027** | -20.083 |
| *90 min less in TV watching* | |  |  |  |  |  |
| Household | 0.0180 | (0.0125, 0.0236) | **-0.0058** | **(-0.0082, -0.0027)** | **0.0086** | -24.448 |
| Sports | 0.0149 | (0.0102, 0.0195) | **-0.0090** | **(-0.0113, -0.0061)** | **0.0000** | -37.727 |
| Work/school | 0.0166 | (0.0114, 0.0218) | **-0.0072** | **(-0.0095, -0.0041)** | **0.0008** | -30.381 |
| Leisure/commute | 0.0169 | (0.0117, 0.0221) | **-0.0069** | **(-0.0091, -0.0040)** | **0.0009** | -29.013 |
| Sleep | 0.0166 | (0.0113, 0.0218) | **-0.0073** | **(-0.0097, -0.0038)** | **0.0020** | -30.449 |
| *120 min less in TV watching* | |  |  |  |  |  |
| Household | 0.0146 | (0.0094, 0.0199) | **-0.0092** | **(-0.0122, -0.0048)** | **0.0034** | -38.654 |
| Sports | 0.0119 | (0.0076, 0.0162) | **-0.0120** | **(-0.0145, -0.0081)** | **0.0000** | -50.164 |
| Work/school | 0.0134 | (0.0085, 0.0182) | **-0.0105** | **(-0.0132, -0.0063)** | **0.0004** | -43.955 |
| Leisure/commute | 0.0136 | (0.0088, 0.0185) | **-0.0102** | **(-0.0129, -0.0060)** | **0.0005** | -42.928 |
| Sleep | 0.0132 | (0.0083, 0.0181) | **-0.0106** | **(-0.0135, -0.0060)** | **0.0010** | -44.501 |

### Older adults

Supplementary Table 17. 1:many reallocations of TV watching time with the rest of the activities in older adults

|  | Predicted prob | 95% CI | Mean difference | 95% CI | P-value | % likelihood in reduction |
| --- | --- | --- | --- | --- | --- | --- |
| Composition without reallocation | 0.0101 | (0.0013, 0.0188) | - | - | - | - |
| 30 min/day | 0.0095 | (0.0011, 0.0179) | -0.0006 | (-0.0016, 0.0004) | 0.3351 | -5.777 |
| 60 min/day | 0.0087 | (0.0006, 0.0168) | -0.0014 | (-0.0032, 0.0010) | 0.2957 | -13.817 |
| 90 min/day | 0.0079 | (0.0000, 0.0157) | -0.0022 | (-0.0048, 0.0015) | 0.2828 | -21.776 |
| 120 min/day | 0.0067 | (0.0000, 0.0144) | -0.0033 | (-0.0064, 0.0026) | 0.2664 | -32.865 |

Supplementary Table 18. 1:1 reallocations of TV-watching in different amounts of time in older adults

|  | Predicted prob | 95% CI | Mean difference | 95% CI | P-value | % likelihood in reduction |
| --- | --- | --- | --- | --- | --- | --- |
| Composition without reallocation | 0.0101 | (0.0013, 0.0188) | - | - | - | - |
| *30 min less in TV watching* | |  |  |  |  |  |
| Household | 0.0097 | (0.0012, 0.0183) | -0.0003 | (-0.0015, 0.0009) | 0.4596 | -3.032 |
| Sports | 0.0071 | (0.0007, 0.0135) | **-0.0030** | **(-0.0048, -0.0011)** | **0.0343** | -29.429 |
| Work/school | 0.0118 | (0.0012, 0.0224) | 0.0018 | (-0.0010, 0.0051) | 0.3395 | 17.491 |
| Leisure/commute | 0.0088 | (0.0011, 0.0166) | -0.0012 | (-0.0022, -0.0002) | 0.0974 | -11.989 |
| Sleep | 0.0095 | (0.0011, 0.0180) | -0.0005 | (-0.0017, 0.0005) | 0.3778 | -5.274 |
| *60 min less in TV watching* | |  |  |  |  |  |
| Household | 0.0091 | (0.0007, 0.0174) | -0.0010 | (-0.0030, 0.0018) | 0.3992 | -9.916 |
| Sports | 0.0063 | (0.0003, 0.0123) | **-0.0037** | **(-0.0059, -0.0013)** | **0.0323** | -37.170 |
| Work/school | 0.0112 | (0.0006, 0.0217) | 0.0011 | (-0.0020, 0.0055) | 0.4603 | 10.944 |
| Leisure/commute | 0.0079 | (0.0007, 0.0151) | -0.0022 | (-0.0037, 0.0000) | 0.1077 | -21.546 |
| Sleep | 0.0088 | (0.0005, 0.0170) | -0.0013 | (-0.0034, 0.0013) | 0.3376 | -12.905 |
| *90 min less in TV watching* | |  |  |  |  |  |
| Household | 0.0083 | (0.0001, 0.0165) | -0.0017 | (-0.0044, 0.0027) | 0.3663 | -17.183 |
| Sports | 0.0056 | (0.0000, 0.0114) | **-0.0044** | **(-0.0068, -0.0011)** | **0.0412** | -43.810 |
| Work/school | 0.0103 | (0.0000, 0.0208) | 0.0003 | (-0.0034, 0.0061) | 0.5207 | 2.943 |
| Leisure/commute | 0.0070 | (0.0000, 0.0139) | -0.0030 | (-0.0051, 0.0002) | 0.1222 | -30.138 |
| Sleep | 0.0080 | (0.0000, 0.0161) | -0.0021 | (-0.0049, 0.0020) | 0.3216 | -20.555 |
| *120 min less in TV watching* | |  |  |  |  |  |
| Household | 0.0072 | (0.0000, 0.0153) | -0.0028 | (-0.0059, 0.0039) | 0.3335 | -28.134 |
| Sports | 0.0048 | (0.0000, 0.0103) | -0.0053 | (-0.0079, -0.0006) | 0.0582 | -52.241 |
| Work/school | 0.0091 | (0.0000, 0.0193) | -0.0010 | (-0.0050, 0.0070) | 0.5133 | -9.948 |
| Leisure/commute | 0.0059 | (0.0000, 0.0125) | -0.0041 | (-0.0066, 0.0013) | 0.1372 | -41.065 |
| Sleep | 0.0069 | (0.0000, 0.0148) | -0.0032 | (-0.0065, 0.0033) | 0.3010 | -31.490 |

## Step 8. Sensitivity analysis

### ≤24 hours of reported movement activities

Since we are working with self-reported data, where sedentary time is often underestimated and physical activity overestimated, we addressed this issue by conducting a sensitivity analysis, including only participants whose total reported time summed to ≤24 hours per day.

In our sample, a total of 45,581 participants (69.6%) had ≤24 hours of reported movement activities.

Supplementary Table 19. Mean composition (proportions) in those with ≤24 hours of reported movement activities

|  | TV watching | Household | Sports | Work/school | Leisure | Sleep |
| --- | --- | --- | --- | --- | --- | --- |
| Total sample  (n=45,581) | 15.88% | 12.82% | 0.30% | 0.26% | 13.00% | 57.73% |

Supplementary Table 20. 1:many reallocations of TV watching time with the rest of the activities in those with ≤24 hours of reported movement activities

|  | Total sample | | | | ≤24 hours | | |
| --- | --- | --- | --- | --- | --- | --- | --- |
|  | Predicted prob | 95% CI | Mean difference | Predicted prob | | 95% CI | Mean difference |
| Composition without reallocation | 0.0238 | (0.0191, 0.0286) |  | 0.0239 | | (0.0182, 0.0296) |  |
| 30 min/day | 0.0229 | (0.0182, 0.0275) | -0.0010 | 0.0231 | | (0.0175, 0.0288) | -0.0008 |
| 60 min/day | 0.0213 | (0.0166, 0.0260) | -0.0026 | 0.0218 | | (0.0160, 0.0277) | -0.0021 |
| 90 min/day | 0.0198 | (0.0150, 0.0247) | -0.0040 | 0.0207 | | (0.0146, 0.0267) | -0.0033 |
| 120 min/day | 0.0177 | (0.0126, 0.0228) | -0.0062 | 0.0189 | | (0.0123, 0.0255) | -0.0050 |

Supplementary Table 21. 1:1 reallocations of TV-watching in different amounts of time in those with ≤24 hours of reported movement activities

|  | Total sample | | | ≤24 hours | | |
| --- | --- | --- | --- | --- | --- | --- |
|  | Predicted prob | 95% CI | Mean difference | Predicted prob | 95% CI | Mean difference |
| Composition without reallocation | 0.0238 | (0.0191, 0.0286) |  | 0.0239 | (0.0182, 0.0296) |  |
| *30 min less in TV watching* | |  |  |  |  |  |
| Household | 0.0234 | (0.0187, 0.0281) | -0.0004 | 0.0237 | (0.0180, 0.0294) | -0.0002 |
| Sports | 0.0205 | (0.0163, 0.0246) | -0.0034 | 0.0206 | (0.0156, 0.0256) | -0.0033 |
| Work/school | 0.0230 | (0.0184, 0.0277) | -0.0008 | 0.0231 | (0.0175, 0.0287) | -0.0008 |
| Leisure/commute | 0.0227 | (0.0181, 0.0272) | -0.0012 | 0.0227 | (0.0172, 0.0281) | -0.0013 |
| Sleep | 0.0228 | (0.0182, 0.0274) | -0.0011 | 0.0231 | (0.0175, 0.0288) | -0.0008 |
| *60 min less in TV watching* | |  |  |  |  |  |
| Household | 0.0220 | (0.0172, 0.0269) | -0.0018 | 0.0226 | (0.0166, 0.0286) | -0.0013 |
| Sports | 0.0188 | (0.0146, 0.0230) | -0.0050 | 0.0192 | (0.0140, 0.0244) | -0.0047 |
| Work/school | 0.0215 | (0.0168, 0.0263) | -0.0023 | 0.0218 | (0.0159, 0.0277) | -0.0021 |
| Leisure/commute | 0.0211 | (0.0165, 0.0256) | -0.0028 | 0.0212 | (0.0156, 0.0268) | -0.0027 |
| Sleep | 0.0211 | (0.0164, 0.0259) | -0.0027 | 0.0218 | (0.0159, 0.0277) | -0.0021 |
| *90 min less in TV watching* | |  |  |  |  |  |
| Household | 0.0207 | (0.0158, 0.0257) | -0.0031 | 0.0216 | (0.0154, 0.0279) | -0.0023 |
| Sports | 0.0174 | (0.0132, 0.0217) | -0.0064 | 0.0181 | (0.0127, 0.0234) | -0.0059 |
| Work/school | 0.0201 | (0.0153, 0.0250) | -0.0037 | 0.0207 | (0.0146, 0.0268) | -0.0032 |
| Leisure/commute | 0.0196 | (0.0149, 0.0243) | -0.0043 | 0.0199 | (0.0142, 0.0257) | -0.0040 |
| Sleep | 0.0197 | (0.0148, 0.0245) | -0.0042 | 0.0206 | (0.0145, 0.0267) | -0.0033 |
| *120 min less in TV watching* | |  |  |  |  |  |
| Household | 0.0186 | (0.0133, 0.0239) | -0.0052 | 0.0199 | (0.0131, 0.0267) | -0.0040 |
| Sports | 0.0155 | (0.0110, 0.0199) | -0.0084 | 0.0164 | (0.0107, 0.0222) | -0.0075 |
| Work/school | 0.0180 | (0.0128, 0.0231) | -0.0059 | 0.0190 | (0.0124, 0.0255) | -0.0049 |
| Leisure/commute | 0.0174 | (0.0125, 0.0224) | -0.0064 | 0.0182 | (0.0120, 0.0244) | -0.0057 |
| Sleep | 0.0175 | (0.0124, 0.0226) | -0.0064 | 0.0189 | (0.0122, 0.0255) | -0.0051 |

### Participants without zero values

In our analyses, we imputed zero values to retain as many participants as possible and avoid loss of statistical power. To assess the impact of this decision, we conducted a sensitivity analysis in which participants with any zero values in movement activities were excluded, resulting in a reduced sample of 18,820 individuals. The table below shows the differences between those included and those excluded under this criterion:

Supplementary Table 22. Differences between participants without and with zero values in movement activities

|  | Without zeros | Including zeros | p-value |
| --- | --- | --- | --- |
|  | n= 18,820 | n= 39,168 |  |
| Age at baseline (mean (SD)) | 42.68 ± 11.18 | 46.11 ± 12.46 | <0.001 |
| Sex (men) (%) | 7114 (37.8) | 16251 (41.5) | <0.001 |
| Education (%) |  |  | <0.001 |
| Low | 3414 (18.1) | 11797 (30.1) |  |
| Middle | 7675 (40.8) | 15925 (40.7) |  |
| High | 7731 (41.1) | 11446 (29.2) |  |
| Equivalized household income (%) |  |  | <0.001 |
| <1230 | 3570 (19.0) | 8648 (22.1) |  |
| ≥1230 to <1590 | 3502 (18.6) | 7697 (19.7) |  |
| ≥1590 to <1900 | 5230 (27.8) | 8769 (22.4) |  |
| ≥1900 | 4430 (23.5) | 8221 (21.0) |  |
| Not provided information | 2088 (11.1) | 5833 (14.9) |  |
| Employment status\ (%) |  |  | <0.001 |
| Employed | 15507 (82.4) | 27027 (69.0) |  |
| Retired | 773 ( 4.1) | 4140 (10.6) |  |
| Unemployed | 2540 (13.5) | 8001 (20.4) |  |

Supplementary Table 23. Mean composition (proportions) in those without zero values in movement activities

|  | TV watching | Household | Sports | Work/school | Leisure | Sleep |
| --- | --- | --- | --- | --- | --- | --- |
| Total sample (n=18,820) | 9.95% | 12.29% | 7.38% | 23.09% | 12.04% | 35.23% |

Table 24. 1:many reallocations of TV watching time with the rest of the activities in those without zero values in movement activities

|  | Total sample | | | Without zeros in activities | | |
| --- | --- | --- | --- | --- | --- | --- |
|  | Predicted prob | 95% CI | Mean difference | Predicted prob | 95% CI | Mean difference |
| Composition without reallocation | 0.0238 | (0.0191, 0.0286) |  | 0.0198 | (0.0124, 0.0273) |  |
| 30 min/day | 0.0229 | (0.0182, 0.0275) | -0.0010 | 0.0172 | (0.0099, 0.0244) | -0.0027 |
| 60 min/day | 0.0213 | (0.0166, 0.0260) | -0.0026 | 0.0142 | (0.0079, 0.0206) | -0.0056 |
| 90 min/day | 0.0198 | (0.0150, 0.0247) | -0.0040 | 0.0116 | (0.0059, 0.0172) | -0.0083 |
| 120 min/day | 0.0177 | (0.0126, 0.0228) | -0.0062 | 0.0083 | (0.0036, 0.0131) | -0.0115 |

Supplementary Table 25. 1:1 reallocations of TV-watching in different amounts of time in those without zero values in movement activities

|  | Total sample | | | Without zeros in activities | | |  |
| --- | --- | --- | --- | --- | --- | --- | --- |
|  | Predicted prob | 95% CI | Mean difference | Predicted prob | 95% CI | Mean difference | |
| Composition without reallocation | 0.0238 | (0.0191, 0.0286) |  | 0.0198 | (0.0124, 0.0273) |  | |
| *30 min less in TV watching* | |  |  |  |  |  | |
| Household | 0.0234 | (0.0187, 0.0281) | -0.0004 | 0.0174 | (0.0101, 0.0246) | -0.0024 | |
| Sports | 0.0205 | (0.0163, 0.0246) | -0.0034 | 0.0171 | (0.0100, 0.0243) | -0.0027 | |
| Work/school | 0.0230 | (0.0184, 0.0277) | -0.0008 | 0.0172 | (0.0101, 0.0244) | -0.0026 | |
| Leisure/commute | 0.0227 | (0.0181, 0.0272) | -0.0012 | 0.0178 | (0.0104, 0.0251) | -0.0021 | |
| Sleep | 0.0228 | (0.0182, 0.0274) | -0.0011 | 0.0168 | (0.0097, 0.0239) | -0.0030 | |
| *60 min less in TV watching* | |  |  |  |  |  | |
| Household | 0.0220 | (0.0172, 0.0269) | -0.0018 | 0.0146 | (0.0081, 0.0211) | -0.0052 | |
| Sports | 0.0188 | (0.0146, 0.0230) | -0.0050 | 0.0142 | (0.0078, 0.0206) | -0.0056 | |
| Work/school | 0.0215 | (0.0168, 0.0263) | -0.0023 | 0.0144 | (0.0080, 0.0207) | -0.0054 | |
| Leisure/commute | 0.0211 | (0.0165, 0.0256) | -0.0028 | 0.0151 | (0.0085, 0.0218) | -0.0047 | |
| Sleep | 0.0211 | (0.0164, 0.0259) | -0.0027 | 0.0137 | (0.0075, 0.0198) | -0.0061 | |
| *90 min less in TV watching* | |  |  |  |  |  | |
| Household | 0.0207 | (0.0158, 0.0257) | -0.0031 | 0.0120 | (0.0062, 0.0178) | -0.0078 | |
| Sports | 0.0174 | (0.0132, 0.0217) | -0.0064 | 0.0116 | (0.0059, 0.0173) | -0.0082 | |
| Work/school | 0.0201 | (0.0153, 0.0250) | -0.0037 | 0.0117 | (0.0061, 0.0174) | -0.0081 | |
| Leisure/commute | 0.0196 | (0.0149, 0.0243) | -0.0043 | 0.0126 | (0.0065, 0.0186) | -0.0072 | |
| Sleep | 0.0197 | (0.0148, 0.0245) | -0.0042 | 0.0109 | (0.0055, 0.0164) | -0.0089 | |
| *120 min less in TV watching* | |  |  |  |  |  | |
| Household | 0.0186 | (0.0133, 0.0239) | -0.0052 | 0.0088 | (0.0038, 0.0137) | -0.0111 | |
| Sports | 0.0155 | (0.0110, 0.0199) | -0.0084 | 0.0084 | (0.0036, 0.0132) | -0.0114 | |
| Work/school | 0.0180 | (0.0128, 0.0231) | -0.0059 | 0.0085 | (0.0038, 0.0133) | -0.0113 | |
| Leisure/commute | 0.0174 | (0.0125, 0.0224) | -0.0064 | 0.0093 | (0.0041, 0.0144) | -0.0106 | |
| Sleep | 0.0175 | (0.0124, 0.0226) | -0.0064 | 0.0078 | (0.0032, 0.0123) | -0.0120 | |

References

1. Chastin, S. F., Palarea-Albaladejo, J., Dontje, M. L., & Skelton, D. A. (2015). Combined Effects of Time Spent in Physical Activity, Sedentary Behaviors and Sleep on Obesity and Cardio-Metabolic Health Markers: A Novel Compositional Data Analysis Approach. PloS one, 10(10), e0139984. <https://doi.org/10.1371/journal.pone.0139984>
2. Pedišić, Ž. (2014). Measurement issues and poor adjustments for physical activity and sleep undermine sedentary behaviour research—the focus should shift to the balance between sleep, sedentary behaviour, standing and activity. *Kinesiology, 46*, 135-146.
3. Mekary, R. A., Lucas, M., Pan, A., Okereke, O. I., Willett, W. C., Hu, F. B., & Ding, E. L. (2013). Isotemporal substitution analysis for physical activity, television watching, and risk of depression. *American journal of epidemiology*, *178*(3), 474–483. <https://doi.org/10.1093/aje/kws590>
4. Rasmussen, C.L., Palarea-Albaladejo, J., Johansson, M.S. *et al.* Zero problems with compositional data of physical behaviors: a comparison of three zero replacement methods. *Int J Behav Nutr Phys Act* **17**, 126 (2020). <https://doi.org/10.1186/s12966-020-01029-z>
5. Palarea-Albaladejo J, Martín-Fernández JA. zCompositions — R package for multivariate imputation of left-censored data under a compositional approach. Chemom Intell Lab Syst. 2015;143:85–96.
6. Mize, T. D. (2019). Best practices for estimating, interpreting, and presenting nonlinear interaction effects. *Sociological Science, 6*(4), 81–117. <https://doi.org/10.15195/v6.a4>
